# Self-Reported Oral Manifestations and Preventive Dental-Care Practices Among Pregnant Women in Romania: A Cross-Sectional Study

**DOI:** 10.3390/jcm15197615

**Published:** 2026-10-01

**Authors:** Ciprian Laurențiu Pătru, Ilona Mihaela Liliac, Dominic Gabriel Iliescu, Anca Mihaela Predescu, Gabriela Boldeanu, Larisa Pătru, Mihaela Jana Țuculină, Alexandra Cătălina Iacov, Cristian Niky Cumpătă, Smaranda Cumpătă, Mihaela Ionescu, Marinas Marius Cristian, Carmen Aurelia Mogoantă, Elena Cristina Andrei

**Affiliations:** 1 Department of Obstetrics and Gynecology, Faculty of Medicine, University of Medicine and Pharmacy of Craiova, 200349 Craiova, Romania; patru.ciprianl@gmail.com (C.L.P.); dominic.iliescu@yahoo.com (D.G.I.); 2 Department of Histology, Faculty of Medicine, University of Medicine and Pharmacy of Craiova, 200349 Craiova, Romaniaelena.andrei@umfcv.ro (E.C.A.); 3 Research Centre for Microscopic Morphology and Immunology, University of Medicine and Pharmacy of Craiova, 200349 Craiova, Romania; anca.predescu@umfcv.ro (A.M.P.); carmen.mogoanta@umfcv.ro (C.A.M.); 4 Department of Histology, Faculty of Dentistry, University of Medicine and Pharmacy of Craiova, 200349 Craiova, Romania; 5 Doctoral School, University of Medicine and Pharmacy of Craiova, 200349 Craiova, Romania; gabrielaboldeanu@gmail.com (G.B.); catalina.iacov0798@gmail.com (A.C.I.); 6 Department 9, Faculty of Medicine, University of Medicine and Pharmacy of Craiova, 200349 Craiova, Romania; 7 Department of Conservative and Restorative Dentistry, Faculty of Dentistry, University of Medicine and Pharmacy of Craiova, 200349 Craiova, Romania; mtuculina@yahoo.com; 8 Faculty of Dentistry, Titu Maiorescu University, 67A Gheorghe Petrașcu Street, 031593 Bucharest, Romania; smarandacumpata@yahoo.com; 9 Department of Medical Informatics and Biostatistics, Faculty of Dentistry, University of Medicine and Pharmacy of Craiova, 200349 Craiova, Romania; mihaela.ionescu@umfcv.ro; 10 Department of Human Anatomy, Faculty of Medicine, University of Medicine and Pharmacy of Craiova, 200349 Craiova, Romania; cristi_marinas84@yahoo.com; 11 Department of Otorhinolaryngology, Faculty of Medicine, University of Medicine and Pharmacy of Craiova, 200349 Craiova, Romania; 12 Dental Medicine Research Center, University of Medicine and Pharmacy of Craiova, 200349 Craiova, Romania

**Keywords:** pregnancy, oral health, oral hygiene, dental care, gingival bleeding, cross-sectional studies

## Abstract

**Background:** Oral symptoms and preventive dental-care practices during pregnancy remain insufficiently characterized in Romania. This study described self-reported oral manifestations, oral-hygiene practices, and dental attendance and explored their cross-sectional associations with gingival bleeding, dental pain, and perceived oral-health impact. **Methods:** This single-center cross-sectional study included 101 pregnant women recruited at the Ginecho Clinic, Craiova, Romania, between October 2025 and July 2026. Participants completed a 21-item electronic questionnaire in the clinic with clarification available from the medical team. Oral conditions were self-reported/perceived rather than clinically diagnosed. Prevalence ratios (PRs) were estimated using Poisson regression with robust variance; multivariable models simultaneously included preventive behaviors, maternal age, parity, and trimester. **Results:** Gingival bleeding (68.3%), gingival swelling (66.3%), and salivary-flow alterations (57.4%) were frequently reported. Most participants brushed once daily (66.3%), never used interdental cleaning (68.3%), and had not attended a dental visit during pregnancy (81.2%). In adjusted models, brushing at least twice daily was associated with lower prevalence of gingival bleeding (aPR = 0.56; 95% CI: 0.32–0.97) and higher perceived oral-health impact (aPR = 0.47; 95% CI: 0.23–0.99). No preventive behavior was significantly associated with dental pain after simultaneous adjustment. **Conclusions:** Self-reported oral manifestations were common and preventive practices were limited in this convenience sample. These exploratory associations do not establish temporality or causality and require confirmation in prospective studies using validated instruments and clinical oral examinations.

## 1. Introduction

### Pregnancy, Oral Health, and Study Rationale

Pregnancy involves endocrine, vascular, immune, and metabolic adaptations that may modify periodontal and oral-tissue responses. Estrogen and progesterone influence vascular permeability and immune regulation, while hCG, relaxin, and prolactin con-tribute to angiogenesis, tissue remodeling, and systemic adaptation [1,2,3,4,5,6,7]. These mechanisms provide biological plausibility for changes in gingival reactivity during gestation, but they do not establish that pregnancy itself causes any specific oral condition in an individual patient [8,9,10,11,12,13,14,15,16]. These mechanisms provide biological plausibility for changes in gingival reactivity during gestation, but they do not establish that pregnancy itself causes any specific oral condition in an individual patient.

The previous literature describes gingival symptoms, salivary and sensory changes, dental discomfort, and altered oral-health-related quality of life during pregnancy [17,18,19,20]. Hormonal influences may increase gingival susceptibility in the presence of plaque and other local factors [21], while oral-hygiene behavior, beliefs about treatment safety, and use of dental services vary considerably between populations [20,22]. A recent multicenter Italian cross-sectional study documented limited dental attendance and pregnancy-related oral complaints, illustrating the need for comparable country-specific data [23]. Romanian evidence on self-reported oral manifestations and preventive dental-care practices during pregnancy remains limited.

Accordingly, the descriptive objective was to estimate the prevalence of self-reported oral manifestations, oral-hygiene practices, and dental attendance in pregnant women attending a clinic in Craiova, Romania. The analytical objective was to explore associations of toothbrushing, interdental cleaning, and dental attendance with three prespecified self-reported outcomes: gingival bleeding, dental pain, and higher perceived impact of pregnancy on oral health. We hypothesized that more frequent preventive oral-health behaviors would be associated with lower prevalence of these outcomes; given the cross-sectional design, all analyses were interpreted as associative rather than causal.

## 2. Materials and Methods

### 2.1. Study Design and Participants

**Figure 1:**
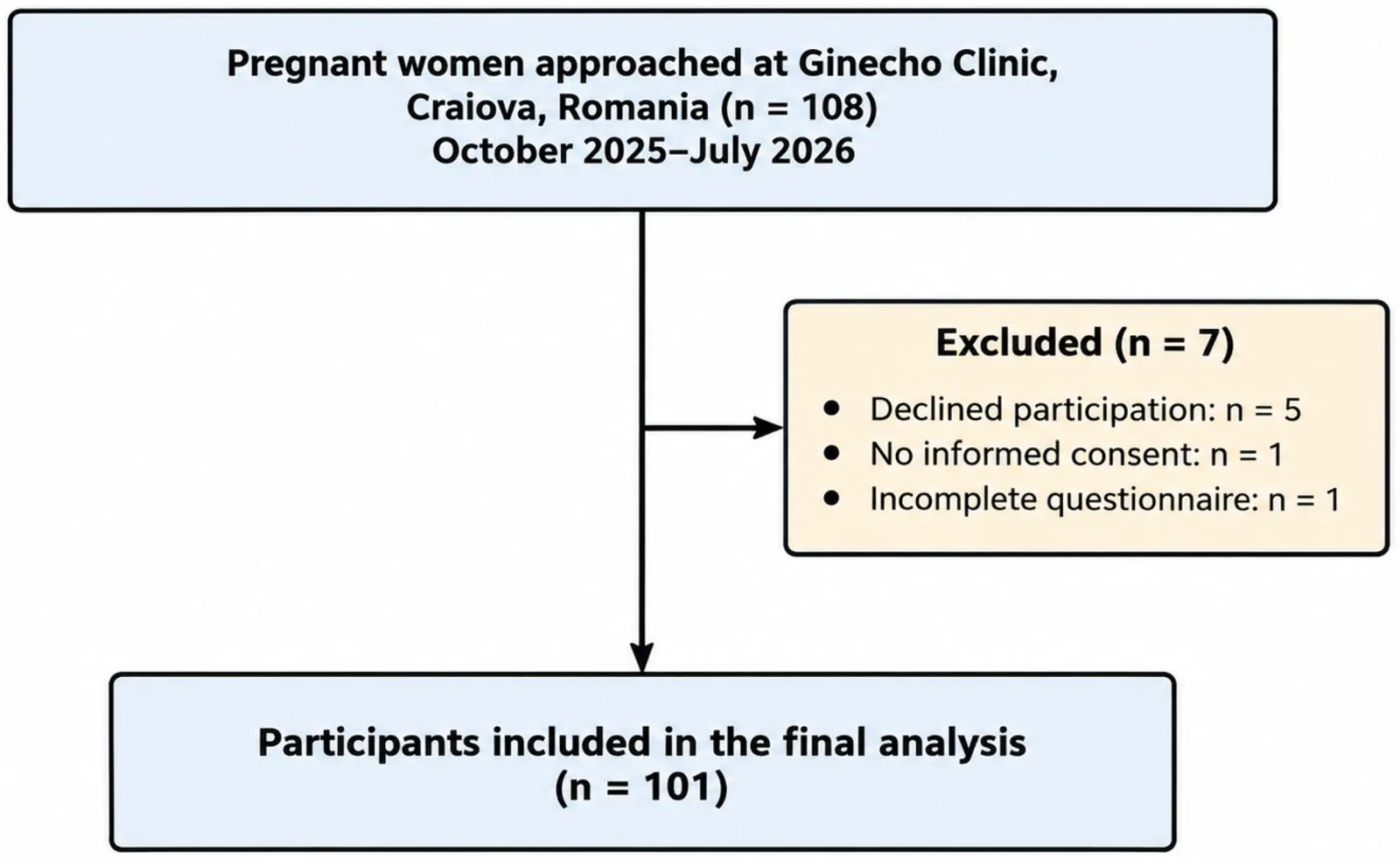
Flow diagram of participant recruitment and inclusion.

A single-center cross-sectional observational study was conducted at the Ginecho Clinic in Craiova, Romania, from October 2025 to July 2026. The source population comprised pregnant women attending the clinic during the study period. Recruitment used a convenience approach: potentially eligible women were invited in the clinic to complete an electronic Google Forms questionnaire in the presence of the medical team. A nurse and physician were available throughout completion to explain the study, clarify terminology, and resolve questions without directing participants’ responses. Of 108 women approached, 101 were included in the analysis (participation/inclusion rate, 93.5%); seven were excluded: five declined participation, one did not provide informed consent, and one submitted an incomplete questionnaire. The participant flow is shown in Figure 1.

Eligible participants were women aged 18 years or older with an ongoing pregnancy clinically confirmed in routine obstetric care and classified as first, second, or third trimester. Exclusion criteria were age < 18 years, ectopic pregnancy or pregnancy loss, absence of informed consent, refusal to participate, and incomplete questionnaire data. Because the questionnaire was administered to women attending pregnancy care, pregnancy status was verified by the clinical team rather than established by the questionnaire itself.

The study is reported with reference to the STROBE recommendations for cross-sectional studies. The recruitment strategy and single-center setting should be considered when assessing representativeness; the sample was not probability-based and no claim of population representativeness is made.

### 2.2. Questionnaire Development

A 21-item questionnaire comprising multiple-choice items and open-ended response fields was developed specifically for this study after review of the literature on oral health during pregnancy. It was an ad hoc study instrument and was not a previously validated psychometric scale. Its purpose was descriptive and exploratory rather than diagnostic.

Before administration, the questionnaire underwent expert review by a five-member multidisciplinary panel from the University of Medicine and Pharmacy of Craiova, Romania comprising one dentist, two obstetricians–gynecologists, one legal specialist, and one biostatistics specialist. The review addressed clinical relevance, clarity, comprehensibility, response structure, and content coverage. Minor linguistic and structural changes were made following this review. Formal psychometric validation, test–retest reproducibility, and quantitative content-validity indices were not performed and are acknowledged as limitations. During administration, medical staff explained all clinical terms requested by participants, including terms referring to perceived carious lesions/restoration failure, oral-mucosal lesions, and pregnancy-associated gingival lesions. These responses therefore represent participant-reported perceptions and symptoms and must not be interpreted as clinical diagnoses. No separate pilot or cognitive pretesting study was performed before recruitment. The complete questionnaire, including the wording of all items and response options, is provided as Supplementary Material S1.

The final questionnaire contained four sections: sociodemographic characteristics; pregnancy-related characteristics; oral-hygiene and dental-care practices; and self-reported oral manifestations and perceived oral-health impact.

Sociodemographic variables included age, residence, and educational level. Pregnancy-related variables included current trimester, number of pregnancies, medical conditions during pregnancy, medication use, and nausea/vomiting. Residence and educational level were not collected through the Google Forms questionnaire; these variables were abstracted from the participants’ clinical observation records maintained at Ginecho Clinic, Craiova, Romania, where questionnaire recruitment and data collection were conducted.

Preventive exposures were self-reported toothbrushing frequency, interdental cleaning frequency, and attendance at a dental examination during the current pregnancy. Unless otherwise specified, these variables referred to participants’ current practices during the ongoing pregnancy.

The three prespecified principal outcomes were self-reported gingival bleeding, self-reported dental pain, and higher self-perceived impact of pregnancy on oral health. Oral manifestations were reported as having occurred during the current pregnancy; for participants reporting a manifestation, trimester of first onset was recalled retrospectively. Because women entered the study at different gestational ages, trimester-of-onset data reflect unequal observation opportunities and retrospective recall and were interpreted descriptively rather than as comparable trimester-specific risks.

Other questionnaire items asked about perceived tooth hypersensitivity, new carious lesions or restoration failure, enamel changes, oral-mucosal lesions, salivary-flow alterations, taste disturbances, and pregnancy-associated gingival lesions. No clinical oral examination was performed; consequently, terms that ordinarily require professional diagnosis are reported throughout as perceived or self-reported conditions. Specifically, the oral-mucosal-lesion item asked participants whether they had noticed “lesions (red/white patches, ulcerations, or growths) of the oral mucosa,” with response options indicating no occurrence or the trimester in which the manifestation was first noticed.

### 2.3. Data Collection

Questionnaires were completed electronically through Google Forms in the clinic under the logistical supervision of the medical team. Responses were reviewed for completeness and eligibility by the study team at the time of collection, which also reduced the possibility of duplicate or unsupervised submissions. The form was administered through a personal account held by a study dentist. Participants’ email addresses were not collected. Access to the original Google Forms responses and to the study dataset was restricted to members of the medical research team involved in the study, and no direct identifiers were included in the analytical dataset. The original electronic study data will be retained under restricted access for one year after publication of the study and will then be deleted in accordance with the study’s data-governance procedures. The use of Google Forms and the specific one-year post-publication retention period were not explicitly specified in the protocol approved by the Ethics Committee; these operational data-governance procedures were implemented by the research team to support confidentiality, controlled access, purpose limitation, and restricted use of the health-related study data.

### 2.4. Statistical Analysis

Data were analyzed using IBM SPSS Statistics version 29.0 (IBM Corp., Armonk, NY, USA). Continuous variables were summarized using mean ± standard deviation, median, interquartile range, and range; categorical variables were summarized as n (%). All 101 included questionnaires were complete for the variables analyzed, and complete-case analysis was therefore used.

The primary descriptive outcomes were the prevalences of self-reported gingival bleeding, dental pain, and higher self-perceived impact of pregnancy on oral health. Prevalence estimates are presented with 95% confidence intervals (CIs). Associations between categorical variables were explored using Pearson’s chi-square test or Fisher’s exact test, as appropriate; exact testing was preferred for sparse contingency tables.

Because gingival bleeding and higher perceived oral-health impact were common outcomes, prevalence ratios (PRs), rather than odds ratios, were used for the principal association analyses. PRs and 95% CIs were estimated using Poisson regression with robust (sandwich) variance. To avoid unstable zero-cell estimates, toothbrushing was prespecified for regression as once daily versus at least twice daily, combining the small ≥3-times/day category (n = 4) with twice-daily brushing. Interdental cleaning was entered as never, occasional, and daily, with never as the reference category.

For each principal outcome, one parsimonious multivariable robust Poisson model simultaneously included toothbrushing frequency, interdental cleaning, dental attendance during pregnancy, maternal age, parity (first versus subsequent pregnancy), and current trimester (first trimester as reference). For the revised analysis, these covariates were selected on clinical and substantive grounds to reduce residual confounding while limiting model complexity in the available sample. Collinearity diagnostics showed variance inflation factors below 3 for all modeled predictors. Education was not included in these models because the participant-level education variable was not present in the analytical spreadsheet available for the regression reanalysis, although the aggregate educational distribution from the original study records is reported descriptively in Table 1; this restriction is acknowledged as a limitation. The numbers of participants with each principal outcome were 69 for gingival bleeding, 28 for dental pain, and 67 for higher perceived oral-health impact. In particular, the dental-pain model was based on only 28 outcome events; therefore, its adjusted estimates, especially those with wide confidence intervals, were interpreted as exploratory and potentially imprecise. No automated variable-selection procedure was used, and no observations were excluded on the basis of influence diagnostics. Because robust Poisson regression was used with binary outcomes specifically to estimate prevalence ratios, inference was based on sandwich-robust standard errors rather than on the conventional Poisson mean-variance assumption; conventional Poisson goodness-of-fit statistics were therefore not used as evidence of binary-outcome model adequacy. Residence and education were available from the clinic observation records for descriptive reporting but had not been merged into the participant-level questionnaire spreadsheet used for the regression reanalysis; consequently, they could not be included in the revised multivariable models.

The ordinal six-level perceived-impact response was additionally examined in a proportional-odds ordinal logistic sensitivity analysis to assess whether conclusions depended on dichotomization. All tests were two-sided with *p* < 0.05 used as a conventional threshold. Because multiple associations were examined in an exploratory single-center sample, *p*-values were not interpreted as confirmatory evidence and emphasis was placed on effect estimates and 95% CIs.

### 2.5. Ethical Considerations

The study was conducted in accordance with the Declaration of Helsinki and was approved by the Ethics Committee of the University of Medicine and Pharmacy of Craiova (Approval No. 108/27.01.2025). Electronic informed consent was obtained before questionnaire completion.

The questionnaire collected health-related information but no participant email addresses or other direct identifiers used in the analytical dataset. Access to the data was limited to the research team. The study therefore used a de-identified research dataset; the term ‘anonymous’ is avoided where irreversible anonymization of all platform-level metadata cannot be demonstrated.

Data handling followed the principles of purpose limitation, data minimization, confidentiality, and restricted access applicable to health information under Regulation (EU) 2016/679 [24]. These safeguards concern data governance and should not be interpreted as evidence regarding participants’ access to dental care or compliance by clinicians with other legal obligations.

## 3. Results

Participant flow is presented first in Figure 1. Of 108 women approached at the clinic, seven were excluded: five declined participation, one did not provide informed consent, and one submitted an incomplete questionnaire, leaving 101 participants for complete-case analysis. Mean age was 30.0 ± 6.2 years (median 30; range 20–44); 78.2% lived in urban areas and 81.2% had higher education. The gestational distribution was unbalanced: 10.9% were in the first trimester, 24.8% in the second, and 64.4% in the third (Table 1).

Preventive practices were limited in this sample. Toothbrushing once daily was reported by 66.3%, twice daily by 29.7%, and ≥3 times/day by 4.0%. Interdental cleaning was never used by 68.3%, while 15.8% reported daily use. Only 18.8% reported a dental visit during the current pregnancy (Table 2).

Self-reported oral manifestations were common (Table 3). Gingival bleeding was reported by 69/101 participants (68.3%; 95% CI: 58.7–76.6%), gingival swelling by 66.3%, perceived salivary-flow alterations by 57.4%, perceived oral-mucosal lesions by 38.6%, taste disturbances by 34.7%, tooth hypersensitivity by 31.7%, and dental pain by 28/101 (27.7%; 95% CI: 19.9–37.1%). The retrospectively reported trimester of first onset of each manifestation is presented in Table 4. A moderate, great, or very great perceived impact of pregnancy on oral health was reported by 67/101 participants (66.3%; 95% CI: 56.7–74.8%; Table 5). These were questionnaire-based self-reports and should not be interpreted as clinically diagnosed disease prevalence.

Unadjusted cross-tabulations are presented as exploratory descriptive comparisons for self-reported gingival bleeding (Table 6), dental pain (Table 7), dental attendance during pregnancy (Table 8), and higher self-perceived impact of pregnancy on oral health (Table 9). For the principal inferential analysis, robust Poisson regression was used to estimate prevalence ratios because odds ratios can materially magnify associations when outcomes are common. Sparse categories and zero cells were not interpreted as evidence of absence of an outcome; instead, clinically related brushing categories were combined for regression as described in Methods.

In the multivariable robust Poisson models that simultaneously included preventive behaviors, age, parity, and trimester (Table 10), brushing at least twice daily was associated with a lower prevalence of self-reported gingival bleeding (adjusted PR [aPR] = 0.56; 95% CI: 0.32–0.97; *p* = 0.039) and higher perceived oral-health impact (aPR = 0.47; 95% CI: 0.23–0.99; *p* = 0.048). Dental attendance showed an inverse association with gingival bleeding, but the CI included the null (aPR = 0.40; 95% CI: 0.14–1.10; *p* = 0.076). None of the three preventive behaviors were statistically associated with dental pain after simultaneous adjustment. These estimates describe cross-sectional prevalence associations and do not establish temporal or causal effects.

In the ordinal sensitivity analysis retaining all six perceived-impact categories, brushing at least twice daily and dental attendance were associated with lower odds of being in a higher perceived-impact category (*p* = 0.017 and *p* = 0.036, respectively), whereas the interdental-cleaning terms were not statistically significant. This sensitivity analysis supports cautious interpretation of the perceived-impact finding while also showing that results depend partly on outcome specification. All inferential analyses remain exploratory because of the sample size, multiple comparisons, and observational design; particular caution is warranted for the dental-pain model, which included only 28 events and yielded several wide confidence intervals.

Frequent nausea/vomiting is a systemic pregnancy symptom rather than a strictly oral manifestation; it is reported alongside the oral manifestations above because repeated vomiting exposes the dentition to gastric acid, which can promote enamel erosion and increase tooth sensitivity, and was therefore considered relevant to participants’ dental health.

Participants who reported a given oral manifestation were additionally asked to indicate the pregnancy trimester in which it had first appeared. As most participants completed the questionnaire during the third trimester, the values below reflect retrospective recall of symptom onset rather than the current-trimester prevalence shown in Table 6 and Table 7, which cross-tabulate current gestational trimester with current symptom status.

Values represent retrospective self-reports of the trimester of first onset for each manifestation among participants who reported experiencing it; they do not represent the number of participants currently in each trimester who have the manifestation (compare with Table 6 and Table 7). Because most participants were surveyed during the third trimester, first-trimester onset values are based predominantly on retrospective recall rather than concurrent report, which may introduce recall bias.

Higher perceived impact was defined as reporting that pregnancy affected oral health “to a moderate extent” or more; lower perceived impact comprised “not at all,” “to a very small extent,” or “to a small extent” (derived from Table 5).

Adjusted prevalence ratios (aPRs) were estimated using Poisson regression with robust (sandwich) variance. Each outcome-specific model simultaneously included all variables shown. Reference categories were toothbrushing once daily, no interdental cleaning, no dental visit during pregnancy, first pregnancy, and first trimester. Maternal age was modeled continuously per one-year increase. Higher self-perceived impact was defined as reporting a moderate, great, or very great impact. All 101 participants had complete data for the variables included in these models. Because the analyses were exploratory, effect estimates and 95% confidence intervals should be emphasized; the dental-pain model included only 28 outcome events and several estimates were imprecise.

## 4. Discussion

In this single-center sample, self-reported gingival bleeding (68.3%) and gingival swelling (66.3%) were common, while 66.3% brushed once daily, 68.3% never used interdental cleaning, and 81.2% had not attended a dental examination during the current pregnancy. In multivariable robust Poisson models simultaneously including preventive behaviors, maternal age, parity, and current trimester, brushing at least twice daily was associated with a lower prevalence of self-reported gingival bleeding (aPR = 0.56; 95% CI: 0.32–0.97) and a lower prevalence of higher self-perceived oral-health impact (aPR = 0.47; 95% CI: 0.23–0.99). None of the preventive behaviors was significantly associated with dental pain after simultaneous adjustment, and the confidence interval for the association between dental attendance and gingival bleeding included the null. These findings describe cross-sectional associations among self-reported variables and do not establish that pregnancy caused the manifestations or that preventive practices prevented them.

The high frequency of perceived gingival symptoms is biologically plausible because pregnancy-related endocrine and vascular changes may modify gingival inflammatory responses, particularly in the presence of local plaque [17,18,19,20,21]. However, no periodontal examination was performed, and self-reported bleeding or swelling cannot be equated with a diagnosis of gingivitis or periodontitis. Likewise, perceived carious lesions, restoration failure, oral-mucosal lesions, and pregnancy-associated gingival lesions were questionnaire responses rather than clinically confirmed conditions.

Our findings are broadly comparable with international questionnaire-based evidence showing limited use of professional dental care and frequent oral complaints during pregnancy [22,23]. The multicenter Italian study by Lo Giudice et al. reported low recent dental attendance and associations between oral-hygiene characteristics and oral symptoms [23]. The present study adds local Romanian data from a clinical setting, but its convenience sampling and single-center design prevent direct population-level inference. Differences between studies may also reflect healthcare organization, questionnaire wording, gestational distribution, socioeconomic characteristics, and oral-health awareness.

Salivary-flow changes, taste disturbances, nausea/vomiting, tooth hypersensitivity, and dental pain were also frequently perceived. Previous studies have described gestational changes in salivary flow and pH [25,26,27,28] and altered taste perception [29]. These mechanisms may help contextualize the reported symptoms, but they cannot verify the individual conditions recorded in this survey. Trimester-of-onset data are particularly vulnerable to recall bias and unequal observation time because most participants were already in the third trimester at enrollment.

The associations with toothbrushing, interdental cleaning, and dental attendance should be interpreted cautiously. Reverse causality is plausible: symptoms may alter hygiene behavior or prompt dental attendance, while women with greater oral-health awareness may both practice preventive behaviors and report symptoms differently. The revised multivariable robust Poisson models simultaneously included the principal preventive behaviors, maternal age, parity, and current trimester; nevertheless, residual confounding by education, socioeconomic circumstances, diet, smoking, pre-existing oral disease, previous dental attendance, and other factors remains possible. The participant-level education variable was not present in the analytical spreadsheet available for the regression reanalysis, although its aggregate distribution is reported descriptively in Table 1. Accordingly, the adjusted prevalence ratios should be interpreted as exploratory associations rather than independent or causal effects. Residence and educational level were obtained from the clinical observation records at Ginecho Clinic rather than from the questionnaire dataset; because these record-derived variables were not linked in the analytical spreadsheet used for the revised regression models, they were not available for multivariable adjustment.

The normative context should be considered separately from the empirical findings of this study. At the European level, the Oviedo Convention establishes general principles concerning patient autonomy, information, and consent in healthcare [30]. Within the Romanian legal framework, Law No. 46/2003 further regulates patients’ rights to information and informed consent [31], while Regulation (EU) 2016/679 provides the applicable framework for the protection and processing of personal and health-related data [24]. These legal instruments provide contextual standards only; the present questionnaire did not assess access barriers, treatment refusal, referral pathways, clinical documentation, information received by patients, or professional legal compliance. Consequently, the low level of dental attendance observed in this sample cannot be interpreted as evidence of a rights violation, professional fault, or a systemic barrier. Questions concerning referral pathways and informed decision-making should therefore be addressed in future studies specifically designed to measure these aspects. Broader oral-health and antenatal-care policy documents may inform interdisciplinary practice [32,33], but such policy considerations remain distinct from the cross-sectional associations observed in the present study.

Overall, the results support the relevance of assessing oral-health experiences during pregnancy while emphasizing the need for clinical confirmation. Prospective, multicenter studies beginning early in pregnancy, using validated instruments and standardized oral examinations, would better establish temporality, reduce recall bias, and clarify whether observed behavioral associations persist after comprehensive confounder adjustment.

### 4.1. Strengths and Limitations of the Study

#### 4.1.1. Strengths

The study provides local Romanian data on an under-described clinical topic and combines information on self-reported oral manifestations, preventive practices, dental attendance, and perceived oral-health impact. Standardized in-clinic electronic administration, predefined eligibility criteria, documented ethical approval, and complete-case analysis of 101 participants are additional practical strengths. The study also enables comparison with recent international questionnaire-based research, while its estimates can inform the design of larger clinically validated studies.

#### 4.1.2. Limitations

Several limitations materially affect interpretation. First, the cross-sectional design precludes temporal and causal inference and permits reverse causality. Participants entered at different gestational stages, so retrospective trimester-of-onset reports reflect unequal observation opportunities and are particularly susceptible to recall bias.

Second, all oral outcomes and preventive behaviors were self-reported. No standardized dental examination verified gingival inflammation, caries, mucosal lesions, restoration failure, or pregnancy-associated gingival lesions. Common-method bias, social-desirability bias, recall error, and differential misclassification are therefore possible. Medical staff were available to explain terminology, which improved comprehension but does not substitute for diagnostic validation.

Third, the 21-item questionnaire was developed for this study and underwent expert review but not formal psychometric validation, quantitative content-validity testing, or reproducibility assessment. The findings should therefore be interpreted as results from an ad hoc descriptive instrument rather than a validated diagnostic or psychometric scale. No separate pilot testing was conducted before study administration.

Fourth, recruitment was a convenience sample from a single clinic. Although 101 of 108 approached women were included, the sample cannot be assumed to represent pregnant women in Craiova or Romania. The trimester distribution was markedly unbalanced (11/25/65), and the predominance of higher education further limits generalizability.

Fifth, although the reanalysis simultaneously adjusted for preventive behaviors, age, parity, and trimester, residual confounding remains possible. Education, socioeconomic status, diet, smoking, pre-existing periodontal disease, previous dental attendance, and other potentially relevant variables were not available for inclusion in the analytical model. The modest sample and sparse cells limited model complexity. Multiple exploratory comparisons were performed without multiplicity correction, increasing the possibility of chance findings.

Sixth, the original odds-ratio analyses could overstate associations for common outcomes. The revised principal analysis therefore used robust Poisson regression to estimate prevalence ratios. Nevertheless, the sample size yields imprecise estimates for several predictors, and the dichotomization of perceived impact entails information loss. An ordinal sensitivity analysis was therefore added, but larger studies with prespecified confounder sets and external validation remain necessary.

Finally, the study did not measure legal compliance, access barriers, costs, treatment refusal, referral quality, documentation, or information received from clinicians. The medical-law discussion is therefore normative and hypothesis-generating, not an empirical finding. The questionnaire was administered through a personal Google Forms account, without collection of participants’ email addresses, and access to the original responses was restricted to the research team. The platform and the specific one-year post-publication retention period were not explicitly prespecified in the ethics-approved protocol. Future studies would benefit from institutionally managed research platforms and prospectively specified data-retention procedures.

## 5. Conclusions

In this single-center cross-sectional convenience sample of 101 pregnant women, self-reported oral manifestations were common and preventive oral-health practices and dental attendance were limited. In multivariable robust Poisson models simultaneously accounting for preventive behaviors, age, parity, and trimester, brushing at least twice daily was associated with lower prevalence of self-reported gingival bleeding and lower prevalence of higher perceived oral-health impact; no preventive behavior was significantly associated with dental pain after adjustment. These exploratory cross-sectional associations do not establish temporality or causality and should be confirmed in larger prospective studies using validated questionnaires and standardized clinical oral examinations.

## Figures and Tables

**Table 1:** Sociodemographic and pregnancy characteristics of the study population.

Variable	n	%
**Total participants**	**101**	
**Age (years)**		
Mean ± SD: 30.0 ± 6.2 years		
Median (IQR): 30 (25–35) years		
Range: 20–44 years		
**Place of residence**		
Urban	79	78.2
Rural	22	21.8
**Educational level**		
Higher education	82	81.2
No higher education	19	18.8
**Current pregnancy**		
First pregnancy	80	79.2
Second pregnancy	14	13.9
Third or subsequent pregnancy	7	6.9
**Pregnancy trimester**		
First trimester	11	10.9
Second trimester	25	24.8
Third trimester	65	64.4
**Medical conditions during pregnancy**		
Yes	2	2.0
No	99	98.0
**Current medication use**		
Yes	23	22.8
No	78	77.2

**Table 2:** Oral hygiene practices during pregnancy.

Variable	n	%
**Toothbrushing frequency**		
Once daily	67	66.3
Twice daily	30	29.7
≥3 times daily	4	4.0
**Dental floss/interdental cleaning**		
Daily	16	15.8
Occasionally	16	15.8
Never	69	68.3
**Dental visit during pregnancy**		
Yes	19	18.8
No	82	81.2

**Table 3:** Oral manifestations reported during pregnancy.

Oral Manifestation	n	%
Frequent nausea/vomiting	68	67.3
Tooth hypersensitivity	32	31.7
New carious lesions/restoration failure	28	27.7
Dental pain	28	27.7
Enamel changes	14	13.9
Gingival bleeding	69	68.3
Gingival swelling	67	66.3
Oral mucosal lesions	39	38.6
Pregnancy tumor	5	5.0
Salivary flow alterations	58	57.4
Taste disturbances	35	34.7

**Table 4:** Trimester of onset of oral manifestations, as retrospectively reported by participants.

Manifestation	First Trimester	Second Trimester	Third Trimester
Tooth hypersensitivity	17	13	2
New caries	15	11	2
Dental pain	14	13	1
Gingival bleeding	14	50	5
Gingival swelling	14	45	8
Oral mucosal lesions	3	27	9
Pregnancy tumor	1	4	0

**Table 5:** Patients’ perception of the impact of pregnancy on oral health.

Response	n	%
Not at all	17	16.8
To a very small extent	1	1.0
To a small extent	16	15.8
To a moderate extent	21	20.8
To a great extent	39	38.6
To a very great extent	7	6.9

**Table 6:** Exploratory crude prevalence-ratio analysis of factors associated with self-reported gingival bleeding during pregnancy.

Variable	Outcome Present n/N (%)	Outcome Absent n/N (%)	Crude PR (95% CI)	*p* Value
**Pregnancy trimester**				0.869
First trimester	7/11 (63.6%)	4/11 (36.4%)	Reference	
Second trimester	18/25 (72.0%)	7/25 (28.0%)	1.13 (0.68–1.88)	
Third trimester	44/65 (67.7%)	21/65 (32.3%)	1.06 (0.66–1.71)	
**Toothbrushing frequency**				<0.001
Once daily	57/67 (85.1%)	10/67 (14.9%)	Reference	
≥2 times/day	12/34 (35.3%)	22/34 (64.7%)	0.41 (0.26–0.66)	
**Interdental cleaning**				<0.001
Never	55/69 (79.7%)	14/69 (20.3%)	Reference	
Occasionally	9/16 (56.2%)	7/16 (43.8%)	0.71 (0.45–1.10)	
Daily	5/16 (31.2%)	11/16 (68.8%)	0.39 (0.19–0.82)	
**Dental visit during pregnancy**				<0.001
No	65/82 (79.3%)	17/82 (20.7%)	Reference	
Yes	4/19 (21.1%)	15/19 (78.9%)	0.27 (0.11–0.64)	
**Medication use**				0.615
No	52/78 (66.7%)	26/78 (33.3%)	Reference	
Yes	17/23 (73.9%)	6/23 (26.1%)	1.11 (0.83–1.48)	

Crude prevalence ratios (PRs) and 95% confidence intervals (CIs) are reported relative to the indicated reference category. Overall *p* values were obtained using Pearson’s chi-square test for multi-category comparisons and Fisher’s exact test for 2 × 2 comparisons. Because the ≥3-times/day brushing category contained only four participants, it was combined with twice-daily brushing as ≥2 times/day to avoid unstable zero-cell estimates. These analyses are exploratory; no multiplicity adjustment was applied, and estimates with wide confidence intervals should be interpreted cautiously.

**Table 7:** Exploratory crude prevalence-ratio analysis of factors associated with self-reported dental pain during pregnancy.

Variable	Dental Pain n/N (%)	No Dental Pain n/N (%)	Crude PR (95% CI)	*p* Value
**Pregnancy trimester**				0.145
First trimester	1/11 (9.1%)	10/11 (90.9%)	Reference	
Second trimester	5/25 (20.0%)	20/25 (80.0%)	2.20 (0.29–16.69)	
Third trimester	22/65 (33.8%)	43/65 (66.2%)	3.72 (0.56–24.88)	
**Toothbrushing frequency**				0.011
Once daily	24/67 (35.8%)	43/67 (64.2%)	Reference	
≥2 times/day	4/34 (11.8%)	30/34 (88.2%)	0.33 (0.12–0.87)	
**Interdental cleaning**				0.018
Never	25/69 (36.2%)	44/69 (63.8%)	Reference	
Occasionally	2/16 (12.5%)	14/16 (87.5%)	0.35 (0.09–1.31)	
Daily	1/16 (6.2%)	15/16 (93.8%)	0.17 (0.03–1.18)	
**Dental visit during pregnancy**				0.261
No	25/82 (30.5%)	57/82 (69.5%)	Reference	
Yes	3/19 (15.8%)	16/19 (84.2%)	0.52 (0.17–1.54)	
**Medication use**				0.599
No	23/78 (29.5%)	55/78 (70.5%)	Reference	
Yes	5/23 (21.7%)	18/23 (78.3%)	0.74 (0.32–1.72)	

Abbreviations, statistical tests, and the combination of toothbrushing categories are as described in the footnote to Table 6.

**Table 8:** Exploratory crude prevalence-ratio analysis of factors associated with dental attendance during pregnancy.

Variable	Dental Visit n/N (%)	No Dental Visit n/N (%)	Crude PR (95% CI)	*p* Value
**Pregnancy trimester**				0.985
First trimester	2/11 (18.2%)	9/11 (81.8%)	Reference	
Second trimester	5/25 (20.0%)	20/25 (80.0%)	1.10 (0.25–4.83)	
Third trimester	12/65 (18.5%)	53/65 (81.5%)	1.02 (0.26–3.93)	
**Current pregnancy**				0.745
First pregnancy	14/80 (17.5%)	66/80 (82.5%)	Reference	
Second pregnancy	3/14 (21.4%)	11/14 (78.6%)	1.22 (0.40–3.72)	
Third or subsequent pregnancy	2/7 (28.6%)	5/7 (71.4%)	1.63 (0.46–5.78)	
**Medication use**				0.131
No	12/78 (15.4%)	66/78 (84.6%)	Reference	
Yes	7/23 (30.4%)	16/23 (69.6%)	1.98 (0.88–4.44)	

Abbreviations, statistical tests, and the combination of toothbrushing categories are as described in the footnote to Table 6.

**Table 9:** Exploratory crude prevalence-ratio analysis of factors associated with a higher self-perceived impact of pregnancy on oral health.

Variable	Higher Perceived Impact n/N (%)	Lower Perceived Impact n/N (%)	Crude PR (95% CI)	*p* Value
**Pregnancy trimester**				0.202
First trimester	5/11 (45.5%)	6/11 (54.5%)	Reference	
Second trimester	19/25 (76.0%)	6/25 (24.0%)	1.67 (0.84–3.31)	
Third trimester	43/65 (66.2%)	22/65 (33.8%)	1.46 (0.74–2.85)	
**Toothbrushing frequency**				<0.001
Once daily	56/67 (83.6%)	11/67 (16.4%)	Reference	
≥2 times/day	11/34 (32.4%)	23/34 (67.6%)	0.39 (0.24–0.64)	
**Interdental cleaning**				<0.001
Never	55/69 (79.7%)	14/69 (20.3%)	Reference	
Occasionally	7/16 (43.8%)	9/16 (56.2%)	0.55 (0.31–0.97)	
Daily	5/16 (31.2%)	11/16 (68.8%)	0.39 (0.19–0.82)	
**Dental visit during pregnancy**				0.001
No	61/82 (74.4%)	21/82 (25.6%)	Reference	
Yes	6/19 (31.6%)	13/19 (68.4%)	0.42 (0.22–0.83)	
**Medication use**				0.805
No	51/78 (65.4%)	27/78 (34.6%)	Reference	
Yes	16/23 (69.6%)	7/23 (30.4%)	1.06 (0.78–1.46)	

Abbreviations, statistical tests, and the combination of toothbrushing categories are as described in the footnote to Table 6.

**Table 10:** Multivariable robust Poisson regression of preventive oral-health behaviors and principal self-reported outcomes.

Outcome	Predictor	aPR	95% CI	*p* Value
Gingival bleeding	Toothbrushing ≥ 2 times/day	0.56	0.32–0.97	0.039
Gingival bleeding	Interdental cleaning, occasional	1.21	0.83–1.77	0.330
Gingival bleeding	Interdental cleaning, daily	1.05	0.47–2.35	0.906
Gingival bleeding	Dental visit during pregnancy	0.40	0.14–1.10	0.076
Gingival bleeding	Maternal age (per year)	1.01	0.99–1.03	0.333
Gingival bleeding	Subsequent vs. first pregnancy	0.90	0.61–1.32	0.586
Gingival bleeding	Second vs. first trimester	1.05	0.65–1.69	0.844
Gingival bleeding	Third vs. first trimester	0.94	0.60–1.46	0.778
Dental pain	Toothbrushing ≥ 2 times/day	0.59	0.15–2.37	0.455
Dental pain	Interdental cleaning, occasional	0.40	0.08–1.99	0.262
Dental pain	Interdental cleaning, daily	0.21	0.03–1.49	0.117
Dental pain	Dental visit during pregnancy	1.71	0.56–5.20	0.342
Dental pain	Maternal age (per year)	1.00	0.95–1.05	0.898
Dental pain	Subsequent vs. first pregnancy	1.17	0.53–2.60	0.703
Dental pain	Second vs. first trimester	1.56	0.22–11.22	0.661
Dental pain	Third vs. first trimester	2.50	0.40–15.78	0.329
Higher self-perceived impact	Toothbrushing ≥ 2 times/day	0.47	0.23–0.99	0.048
Higher self-perceived impact	Interdental cleaning, occasional	1.00	0.56–1.81	0.988
Higher self-perceived impact	Interdental cleaning, daily	0.98	0.38–2.55	0.972
Higher self-perceived impact	Dental visit during pregnancy	0.81	0.35–1.87	0.616
Higher self-perceived impact	Maternal age (per year)	1.02	1.00–1.04	0.028
Higher self-perceived impact	Subsequent vs. first pregnancy	0.64	0.38–1.07	0.086
Higher self-perceived impact	Second vs. first trimester	1.52	0.89–2.59	0.126
Higher self-perceived impact	Third vs. first trimester	1.25	0.75–2.10	0.396

Abbreviations, statistical tests, and the combination of toothbrushing categories are as described in the footnote to Table 6.

## Data Availability

De-identified study data may be made available upon reasonable request to the corresponding author. Requests will be evaluated by the study investigators and, where necessary, the responsible institutional/ethics structures, taking into account the scientific purpose of the request, participant confidentiality, the scope of the original consent, and applicable ethical and data-protection requirements. The dataset is not publicly posted because it contains health-related research information. The original electronic study data are retained under restricted access for one year after publication and are scheduled for deletion thereafter; any controlled-access sharing during that period remains subject to the conditions stated above.

## Supplementary Materials

The following supporting information can be downloaded at: https://www.mdpi.com/article/10.3390/jcm15197615/s1, Supplementary_Material_S1_Questionnaire EN and Romanian.

## References

[1] Parisi F., Fenizia C., Introini A., Zavatta A., Scaccabarozzi C., Biasin M., Savasi V. (2023). The pathophysiological role of estrogens in the initial stages of pregnancy: Molecular mechanisms and clinical implications for pregnancy outcome from the periconceptional period to end of the first trimester. Hum. Reprod. Update.

[2] Schumacher A., Costa S.D., Zenclussen A.C. (2014). Endocrine factors modulating immune responses in pregnancy. Front. Immunol..

[3] Bai J., Qi Q.R., Li Y., Day R., Makhoul J., Magness R.R., Chen D.B. (2020). Estrogen Receptors and Estrogen-Induced Uterine Vasodilation in Pregnancy. Int. J. Mol. Sci..

[4] Wei C., Wu H., Yu Y., Li Y., Xiang S., Lian F. (2024). Effect of estrogen exposure on pregnancy outcomes in artificial frozen-thawed embryo transfer cycles. Gynecol. Endocrinol..

[5] Hizkiyahu R., Suarthana E., Kadour Peero E., Feferkorn I., Buckett W. (2022). Does increasing estrogen dose during frozen embryo transfer affect. pregnancy rate?. J. Assist. Reprod. Genet..

[6] Qin X.D., Zhou Y., Bloom M.S., Qian Z.M., Geiger S.D., Vaughn M.G., Chu C., Li Q.Q., Yang B.Y., Hu L.W. (2023). Prenatal Exposure to PFAS, Associations with Preterm Birth and Modification by Maternal Estrogen Levels: The Maoming Birth Study. Environ. Health Perspect..

[7] Bulletti C., Bulletti F.M., Sciorio R., Guido M. (2022). Progesterone: The Key Factor of the Beginning of Life. Int. J. Mol. Sci..

[8] Zwahlen M., Stute P. (2024). Impact of progesterone on the immune system in women: A systematic literature review. Arch. Gynecol. Obstet..

[9] Skogler J., Moberg T., Tancredi L., Styrmisdottir L., Hedayati E., Alarcon-Ruiz C.A., Khamis A., Persad E., Iskandarani G., Hansson S.R. (2023). Association between human chorionic gonadotropin (hCG) levels and adverse pregnancy outcomes: A systematic review and meta-analysis. Pregnancy Hypertens..

[10] Goldsmith L.T., Weiss G. (2009). Relaxin in human pregnancy. Ann. N. Y. Acad. Sci..

[11] Conrad K.P. (2011). Maternal vasodilation in pregnancy: The emerging role of relaxin. Am. J. Physiol. Regul. Integr. Comp. Physiol..

[12] Dschietzig T., Stangl K. (2003). Relaxin: A pregnancy hormone as central player of body fluid and circulation homeostasis. Cell. Mol. Life Sci..

[13] Henneman S., Bildt M.M., Degroot J., Kuijpers-Jagtman A.M., Von den Hoff J.W. (2008). Relaxin stimulates MMP-2 and alpha-smooth muscle actin expression by human periodontal ligament cells. Arch. Oral Biol..

[14] Rana M., Jain S., Choubey P. (2022). Prolactin and its significance in the placenta. Hormones.

[15] Capozzi A., Scambia G., Pontecorvi A., Lello S. (2015). Hyperprolactinemia: Pathophysiology and therapeutic approach. Gynecol. Endocrinol..

[16] Surarit R., Krishnamra N., Seriwatanachai D. (2016). Prolactin receptor and osteogenic induction of prolactin in human periodontal ligament fibroblasts. Cell Biol. Int..

[17] Silk H., Douglass A.B., Douglass J.M., Silk L. (2008). Oral health during pregnancy. Am. Fam. Physician.

[18] Hartnett E., Haber J., Krainovich-Miller B., Bella A., Vasilyeva A., Lange Kessler J. (2016). Oral Health in Pregnancy. J. Obstet. Gynecol. Neonatal Nurs..

[19] Saadaoui M., Singh P., Al Khodor S. (2021). Oral microbiome and pregnancy: A bidirectional relationship. J. Reprod. Immunol..

[20] Kamalabadi Y.M., Campbell M.K., Zitoun N.M., Jessani A. (2023). Unfavourable beliefs about oral health and safety of dental care during pregnancy: A systematic review. BMC Oral Health.

[21] Mealey B.L., Moritz A.J. (2003). Hormonal influences: Effects of diabetes mellitus and endogenous female sex steroid hormones on the periodontium. Periodontology 2000.

[22] Johnson E.K., Okyere Boadu K.A. (2026). Oral Hygiene Practices, Knowledge, and Awareness of Periodontal Disease Among Pregnant Women Visiting the Antenatal Clinic at Korle-Bu Teaching Hospital. Public Health Chall..

[23] Lo Giudice R., Martinelli C., Alibrandi A., Mondo A., Venezia R., Cannarozzo M.G., Puleio F., Pollicino R., Lo Giudice G., Lagana A.S. (2024). Multicenter Cross-Sectional Study of Oral Health and Hygiene Practices Among Pregnant Women. J. Clin. Med..

[24] European Parliament, Council of the European Union (2016). Regulation (EU) 2016/679 of 27 April 2016 on the protection of natural persons with regard to the processing of personal data and on the free movement of such data (General Data Protection Regulation). Off. J. Eur. Union.

[25] Islam N.A.B., Haque A. (2024). Pregnancy-related dental problems: A review. Heliyon.

[26] Morton A., He J.W. (2025). Ptyalism gravidarum. Obstet. Med..

[27] Migliario M., Bindi M., Surico D., De Pedrini A., Minsenti S., Pezzotti F., Mele B., Foglio Bonda P.L. (2021). Changes in salivary flow rate and pH in pregnancy. Eur. Rev. Med. Pharmacol. Sci..

[28] Yilmaz F., Carti Dorterler O., Eren Halici S., Kasap B., Demirbas A. (2024). The effects of pregnancy on oral health, salivary ph and flow rate. BMC Oral Health.

[29] Nordin S., Broman D.A., Olofsson J.K., Wulff M. (2004). A longitudinal descriptive study of self-reported abnormal smell and taste perception in pregnant women. Chem. Senses.

[30] Council of Europe (1997). Convention for the Protection of Human Rights and Dignity of the Human Being with Regard to the Application of Biology and Medicine: Convention on Human Rights and Biomedicine (Oviedo Convention).

[31] Romania Government (2003). Legea nr. 46/2003 Privind Drepturile Pacientului [Law No. 46/2003 on Patients’ Rights], as Subsequently Amended. Monitorul Oficial al României 2003.

[32] World Health Organization (2024). Global Strategy and Action Plan on Oral Health 2023–2030.

[33] World Health Organization (2016). WHO Recommendations on Antenatal Care for a Positive Pregnancy Experience.

