# Supplementary material for: Self-Reported Oral Manifestations and Preventive Dental-Care Practices Among Pregnant Women in Romania: A Cross-Sectional Study
Source: J Clin Med. 2026 Oct 1;15(19):7615. doi: 10.3390/jcm15197615 (PMC13665922; doi:10.3390/jcm15197615)
Supplement: Supplementary file 1 [file jcm-15-07615-s001.zip › jcm-4586519-supplementary.pdf]

Chestionar de evaluare a sănătății orale în sarcină

* Indică o întrebare obligatorie

1. Varsta *

2. Numar de sarcini anterioare *

3. Sarcina curenta *

Marchează un singur oval.

- Prima
- a doua
- a treia sau mai mult

4. Trimestrul actual al sarcinii *

Marchează un singur oval.

- I
- II
- III

5. 7. Ați fost diagnosticată cu vreo afecțiune medicală în timpul sarcinii? *

Marchează un singur oval.

- da
- Nu

6. Urmați vreun tratament medicamentos în prezent? *

Marchează un singur oval.

- da
- Nu

7. Ați suferit de grețuri sau vărsături frecvente în timpul sarcinii? *

Marchează un singur oval.

- nu
- da trimistrul I
- da trimestrul II
- da trimestrul III

8. Cât de des vă periați dinții? *

Marchează un singur oval.

- o data pe zi
- de doua ori pe zi
- de trei sau mai multe ori

9. Utilizați ață dentară sau duș bucal? *

Marchează un singur oval.

- nu
- ocazional
- zilnic

10. Ați fost la control stomatologic în timpul sarcinii? *

Marchează un singur oval.

- da
- nu

11. Ați observat sensibilitate crescută la nivelul dinților? (se precizeaza trimestrul) *

Marchează un singur oval.

- nu
- da trimestrul I
- da trimestrul II
- da trimestrul III

12. Ați observat carii noi sau deteriorarea unor obturații vechi? (daca da specificați locația aproximativă și trimestrul) *

Marchează un singur oval.

- nu
- da trimestrul I
- da trimestrul II
- da trimestrul III

13. Ați avut dureri dentare în această sarcină? (daca da se precizeaza si trimestrul) *

Marchează un singur oval.

- nu
- da trimestrul I
- da trimestrul II
- da trimestrul III

14. Ați observat modificări de culoare sau aspect la nivelul smalțului? (daca da descrieti) *

Marchează un singur oval.

- nu
- da

15. Ați observat sângerări gingivale la periaj sau spontan? *

Marchează un singur oval.

- nu
- da trimestrul I
- da trimestrul II
- da trimestrul III

16. Ați observat umflarea gingiilor? *

Marchează un singur oval.

- nu
- da trimestrul I
- da trimestrul II
- da trimestrul III

17. Ați observat apariția unor leziuni (pete roșii/albe, ulcerații, excrescențe) la nivelul mucoasei orale? *

Marchează un singur oval.

- nu
- da trimestrul I
- da trimestrul II
- da trimestrul III

18. Ați dezvoltat o excrescență gingivală ("tumora gravidei")? *

Marchează un singur oval.

- nu
- da trimestrul I
- da trimestrul II
- da trimestrul III

19. Ați observat o modificare a fluxului salivar? *

Marchează un singur oval.

- nu
- da- hipersalivatie
- da - gura uscata

20. Ați observat modificări ale gustului? *

Marchează un singur oval.

- nu
- da - gust metalic
- da - gust amar
- da - pierderea gustului

21. Considerati ca sarcina a afectat sanatatea dvs orala? *

Marchează un singur oval.

- In foarte mare masura
- in mare masura
- in masura moderata
- in mica masura
- in foarte mica masura
- deloc
- Opțiunea 7

Acest conținut nu este nici creat, nici aprobat de Google.

Formulare Google
